# Modification of Polyamide-Urethane (PAUt) Thin Film Composite Membrane for Improving the Reverse Osmosis Performance

**DOI:** 10.3390/polym10040346

**Published:** 2018-03-21

**Authors:** Li-Fen Liu, Xing-Ling Gu, Sa-Ren Qi, Xin Xie, Rui-Han Li, Ke Li, Chun-Yang Yu, Cong-Jie Gao

**Affiliations:** 1 Center for Membrane and Water Science and Technology, Ocean College, Zhejiang University of Technology, Hangzhou 310014, China; 13588348159@163.com (X.-L.G.); 15958041452@163.com (X.X.); 15857112095@163.com (R.-H.L.); gaocj@zjut.edu.cn (C.-J.G.); 2 Collaborative Innovation Center of Membrane Separation and Water Treatment of Zhejiang Province, Hangzhou 310014, China; 3 Singapore Membrane Technology Centre, Nanyang Technological University, Singapore 639798, Singapore; qisaren@gmail.com; 4 School of Chemistry & Chemical Engineering, State Key Laboratory of Metal Matrix Composites, Shanghai Jiao Tong University, 800 Dongchuan Road, Shanghai 200240, China; like1990@sjtu.edu.cn (K.L.); chunyangyu@sjtu.edu.cn (C.-Y.Y.)

**Keywords:** poly (amide-urethane), reverse osmosis, layer-by-layer assembly, antifouling

## Abstract

In the current study, the poly (amide-urethane) (PAUt) membranes were successfully fabricated by interfacial polymerization of m-phenylenediamine (MPD) and 5-choroformyloxyisophaloyl chloride (CFIC) on the polysulfone substrates. Two modification methods based on layer-by-layer assembly were applied to modify the PAUt membrane surface to achieve antifouling property: 1. Chitosan (CS) was directly self-assembled on the PAUt membrane (i.e., PAUt-CS); and 2. polydimethyl diallyl ammonium chloride (PDDA), polystyrene sulfonate (PSS), and CS were successively self-assembled on the membrane surface (i.e., PAUt-PDDA/PSS/CS). The resultant membranes were symmetrically characterized by Attenuated Total Reflection Fourier Transform Infrared Spectroscopy (ATR-FTIR), X-ray Photoelectron Spectroscopy (XPS), Scanning Electron Microscopy (SEM), Atomic Force Microscopy (AFM) and Contact Angle Meter (CAM), respectively. The results indicated that the modified membranes had much smoother and more hydrophilic surfaces as compared to the nascent PAUt membrane. Meanwhile, the modified membranes exhibited better reverse osmosis performance in terms of water permeability and salt rejection. After the modified membranes were fouled by lake water, the PAUt-PDDA/PSS/CS membrane presented the best antifouling performance among the three types of membranes. Combining the reverse osmosis performance with the anti-fouling property obviously, the PAUt-PDDA/PSS/CS membrane behaved as a promising candidate to be used in real applications.

## 1. Introduction

Reverse osmosis (RO) membrane is one of the core parts in the process of RO technology, which has been widely used in the process of desalination, wastewater treatment, food production process, etc. [1,2]. Generally, thin film composite (TFC) RO membranes, developed using an interfacial polymerization (IP) technique, have been dominating the RO market because they offer stability in a wider pH range, higher solute rejection, and better resistance to microbial degradation than the preceded cellulose acetate integral membranes. Furthermore, the IP method allows for the performance optimization of the as-formed polyamide layers by designing functional monomers.

The selective separation layer in the TFC RO membrane is commonly fabricated from the interfacial polymerization of m-phenylenediamine (MPD) in aqueous solution and trimesoyl chloride (TMC) in organic solution on a porous polysulfone support membrane [3]. The selective separation layer is critical for the membrane separation properties in terms of water permeability, salt rejection and fouling resistance. Nowadays, roughness induced fouling and free chlorine intolerance remain as big obstacles for the widespread application of RO membranes in terms of long-term stability [4,5,6]. In the efforts to improve the anti-fouling property of the selective separation layer, various techniques were adopted in the literature, such as coating [7], grafting [8], layer by layer (LbL) [9], etc. Among them, the LbL assembly technique appears to be attractive due to its ability to regulate the porosity, thickness, and chemical composition of the membrane surface [10,11,12,13,14,15,16,17,18,19,20,21,22]. Although the LbL technique has been extensively investigated to form the selective separation layer of nanofiltration membrane, forward osmosis membrane, reverse osmosis membrane with multi-layers assembly to achieve salt rejection, less studies report the LbL assembly using a few layers to tune the property of the polyamide selective separation layer for antifouling RO membranes [9,23]. On the other hand, in order to improve the anti-chlorine property of the TFC membrane, many efforts have been explored, such as the development of new monomers, optimization of IP process, and incorporation of nanomaterials, etc. [24,25,26,27,28,29,30,31,32,33,34]. Particularly, in our group, a novel monomer of 5-choroformyloxyisophaloyl chloride (CFIC) was synthesized [34]. The monomer reacted with 4-methyl-phenylenediamine (MMPD) via interfacial polymerization to form a novel poly (amide-urethane) RO membrane. The resultant membrane showed excellent chlorine-tolerance. However, due to the rougher surface of the selective separation layer, the fouling was quite severe for the poly (amid-urethane) RO membranes.

In the current study, we aim to modify the selective separation layer of the poly (amide-urethane) RO membrane to achieve better anti-fouling property. LbL technique was selected to improve the hydrophilicity and roughness of the selective separation layer. Three typical polyelectrolytes were utilized: poly (diallyl dimethyl ammonium chloride) (PDDA), polystyrene sulfonate (PSS), and chitosan (CS). The chemical compositions and morphologies of the resultant membranes were systematically characterized by Attenuated Total Reflectance-Fourier Transform Infrared Spectroscopy (ATR-FTIR), X-ray Photoelectron Spectroscopy (XPS), Scanning Electronic Microscopy (SEM). The membrane surface roughness and hydrophilicity were analyzed by Atomic Force Microscopy (AFM) and contact angle measurement, respectively. Reverse osmosis membrane performance and fouling experiments were conducted using a customized pressure-driven cross-flow filtration setup. To the best knowledge of the authors, it is the first study to perform surface modification of the chlorine resistant polyamide-urethane (PAUt) RO membrane for improved reverse osmosis performance. 

## 2. Materials and Methods

### 2.1. Materials and Reagents

Poly (diallyldimethyl ammonium chloride) (PDDA, M_w_: 1–2 × 10^5^ Da), poly (sodium styrene sulfonate) (PSS, M_w_: 2 × 10^6^ Da), and chitosan (CS, M_w_: 1–2 × 10^5^ Da) were purchased from Sigma Aldrich. 5-choroformyloxyisophaloyl chloride (CFIC, Purity ≥ 99%) was synthesized via tri-phosgene (BTC) method in the presence of composite catalyst imidazole-pyridine according to Ref. [34]. m-phenylenediamine (MPD), triethyl amine (TEA), (+)-10-champhor sulfonic acid (CSA), glutaconaldehyde (GA), and sodium dodecyl sulfate (SDS) were purchased from J&K Scientific Ltd. (Beijing, China) Sodium hydroxide (NaOH), *n*-hexane, and sodium chloride (NaCl) were purchased from Sinopharm Chemical Reagent Co., Ltd. (Shanghai, China). All chemicals were used as received without further purification. Deionized (DI) water with the conductivity less than 2 µs/cm was produced by a two-stage reverse osmosis system.

### 2.2. Fabrication of Polyamide-Urethane (PAUt) Membrane

The polysulfone (PSF) substrates with Mw of 200,000 Da were supplied by the Center of Water Treatment Technology, Hangzhou, China. The PAUt membrane was fabricated by the conventional interfacial polymerization method, which was described in literature [34]. Briefly, the PSF substrate was clamped between two Teflon frames with the inner length and width of 20 cm and 15 cm, respectively. The top surface of PSF substrate was immersed into the aqueous solution (the composition was 2.0 wt% MPD, 2.0 wt% TEA, 0.15 wt% SDS, and 4.0 wt% CSA) for 2 min. The excess solution was then drained, and the membrane was air-dried at ambient temperature until no visible liquid drops were on the surface. Subsequently, the organic solution containing 0.15 wt% CFIC was reacted with residual MPD on the surface of PSF substrate for 60 s. The resultant membrane was finally heated at 100 °C in the air dryer for further polymerization. The obtained PAUt membrane was washed by DI water and stored in 1.0 wt% NaHSO_3_ solution for further modification.

### 2.3. Modification of the Selective Separation Layer of PAUt Membrane

**Figure 1:**
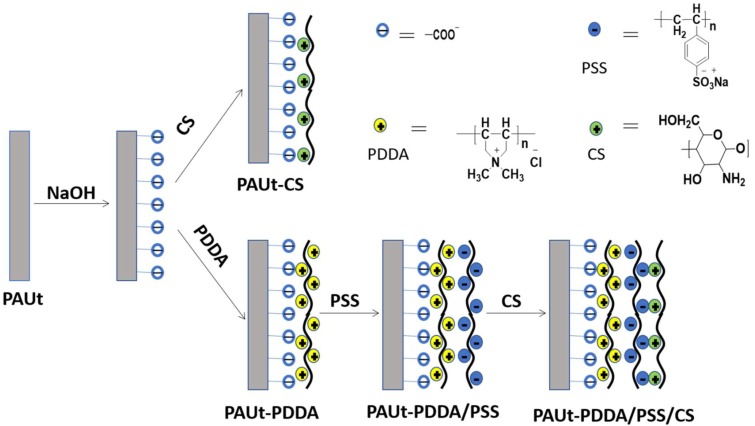
The schematic drawing process for modification of the selective separation layer of PAUt membrane.

The procedure of hydrolyzation was adopted from the Ref. [35]. In brief, the nascent selective separation layer of PAUt membrane was firstly hydrolyzed in NaOH solution (pH = 7–12) for a certain period (i.e., 20 min). Then, DI water was applied to flush the surface until the pH of washing water become neutral. There are two loops to further modify the PAUt membrane (Figure 1). In the first loop, the top surface of the PAUt membrane was directly coated with 0.1–0.5 wt% CS for 20 min. The resultant membrane was labeled as PAUt-CS. In the second loop, the top surface of the PAUt membrane was firstly coated with 0.2 wt% positively charged PDDA for 20 min, after washing with DI water for 5 min, the membrane was then coated with the 0.2 wt% negatively charged PSS for another 20 min. Finally, the membrane was coated with 0.1–0.5 wt% CS for 20 min after DI water rinsing the surface. The resultant membrane was labeled as PAUt-PDDA/PSS/CS. After coating with the polyelectrolytes, 0.2 wt% GA solution was applied to crosslink the polyelectrolytes and the crosslinking process was conducted under 60 °C for 30 min [36].

### 2.4. Characterization of Membrane

The functional groups and bonds of the surface were investigated by FTIR-ATR (Nicolet 6700 FTIR, Thermo Electron Corporation, Waltham, MA, USA) and XPS (Kratos AXIS Ultra DLD, Shimadzu Corporation, Chiyoda-ku, Tokye, Japan), respectively. The membrane surface morphologies were characterized by SEM (S-4700, Hitachi Ltd., Tokyo, Japan) and AFM (SPA-400, Seiko Ltd., Tokyo, Japan) based on the Ref. [37], respectively. Both surface and cross-section were prepared and sputtered the Pt coating before SEM characterization. Membrane surface hydrophilicity was characterized by contact angle meter (CAM) (DSA10-MK2, KRÜSS GmbH, Hamburg Germany) with at least 20 independent measurements. All membrane samples were prepared with removing the NaHSO_3_ solution by thoroughly rinsing with DI water and dried under the freeze-dry conditions to prevent artifact of the membrane.

### 2.5. Reverse Osmosis Membrane Performance

**Figure 2:**
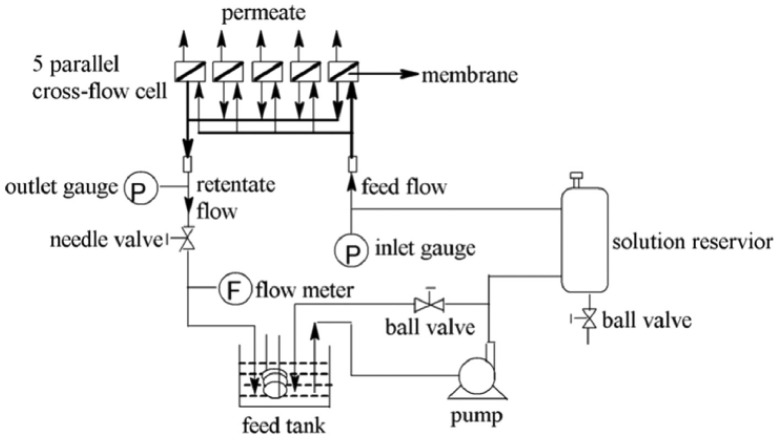
Setup schematic drawing for evaluation of RO performance for the membrane.

Reverse osmosis membrane performances in terms of water flux and salt rejection were performed and evaluated [34]. A cross-flow reverse osmosis testing setup with five parallel cells was used for the current study (Figure 2). Membrane coupons with a diameter of 40 mm with an effective area of 12 cm^2^ were thoroughly washed by DI water prior to loading to the cell. Firstly, the membrane was compacted with DI water under 1.55 MPa for at least 2 h. After flux was maintained steady for at least 30 min, a 2000 ppm NaCl feed solution was applied with a constant cross flow velocity of 8.0 liter per minute (LPM). The water flux Jv was determined by the gravimetric method. The salt rejection R was determined via Equation (1). The conductivity meter (DDS-307, Cany instruments Co. Ltd., Shanghai, China) measured the conductivity of permeate *C*_p_ and conductivity of feed solution *C*_f_, respectively.

(1) R=1−CpCf

The fouling evaluation of the membrane was conducted using the same setup. Each membrane coupon was soaked into the real lake water (i.e., West Lake in Hangzhou, China, Table S2) for seven days. After the surface of the coupon was slightly rinsed with DI water, the membrane filtration performance was evaluated in the same setup and same procedure. All the filtration evaluations (both salt and fouling experiments) were conducted using at least three independent membrane samples. Each measurement was duplicated at least three times. The average results were performed in Section 3.

## 3. Results

The polyamide-urethane (PAUt) membrane was successfully fabricated via MPD and CFIC monomers. Two types of modifications were applied to successfully modify the PAUt selective separation layer for antifouling purpose without scarifying the reverse osmosis performance. Several results were obtained as the following.

### 3.1. Chemical Composition, Physical Morphology and Hydrophilicity of the Membrane Selective Layer

**Figure 3:**
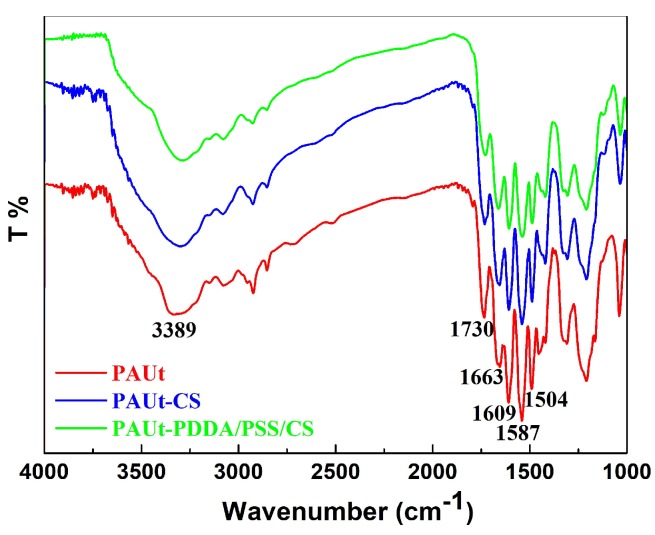
ATR-FTIR spectrum of PAUt, PAUt-CS, and PAUt-PDDA/PSS/CS membrane, respectively.

**Figure 4:**
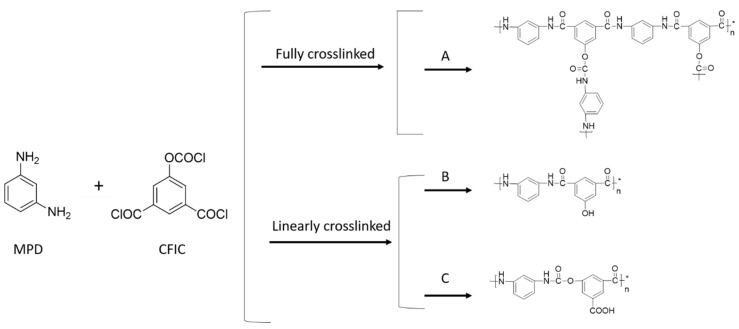
Typical chemistry for interfacially formed PAUt membranes. A to C represented the ideal chemical structure of resultant PAUt selective separation layer formed by MPD and CFIC.

**Figure 5:**
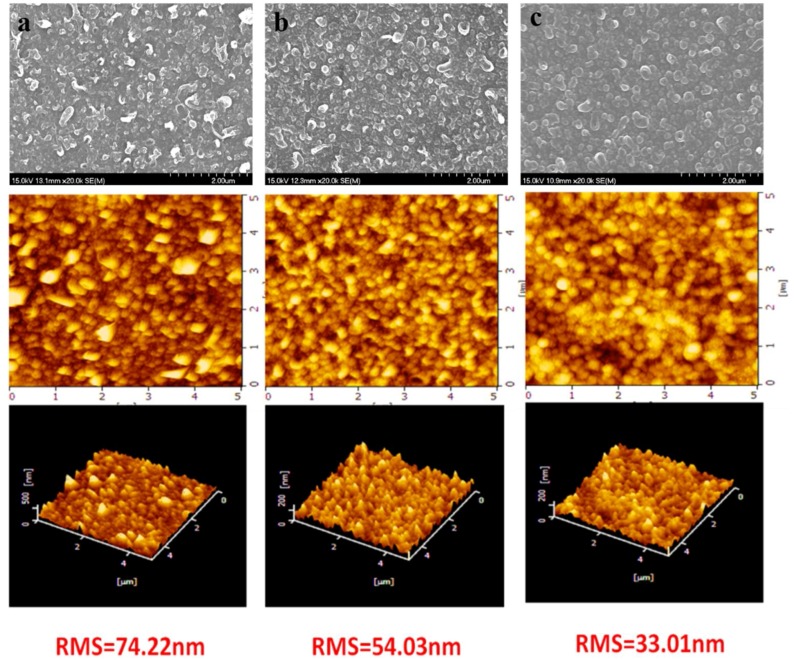
SEM (first line) and AFM images (second and third line) of (**a**) PAUt membrane; (**b**) PAUt-CS membrane and (**c**) PAUt-PDDA/PSS/CS membrane, respectively.

**Figure 6:**
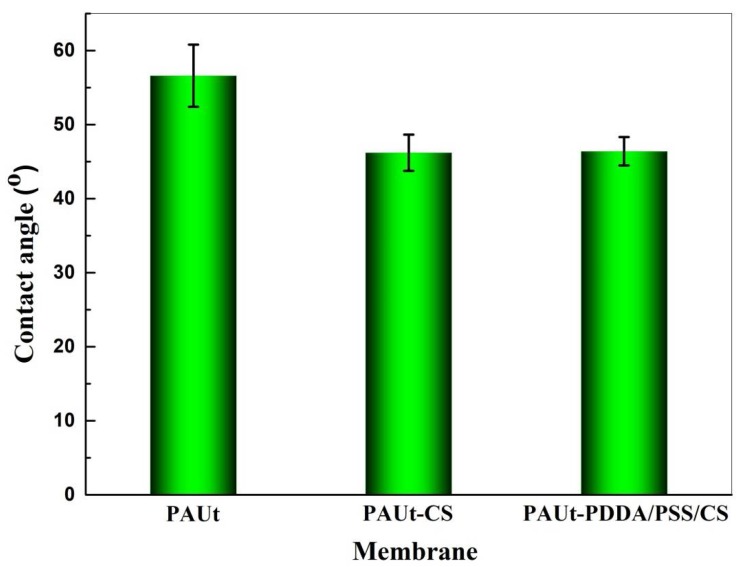
Water contact angle of the membranes.

FTIR-ATR, XPS, FESEM, AFM and contact angle meter were used to characterize systematically the chemical composition and physical morphology of the resultant membranes. The chemical composition of the membrane selective separation layer was analyzed by FTIR-ATR (Figure 3) and XPS (Figure 4 and Table 1) respectively, and the results showed that the PAUt membrane surfaces were successfully modified by CS molecules. Moreover, the two modified membranes of PAUt-CS and PAUt-PDDA/PSS/CS exhibited smoother (Figure 5) and more hydrophilic (Figure 6) surface than the nascent PAUt membrane. 

### 3.2. Reverse Osmosis Performance of the Membranes

**Figure 7:**
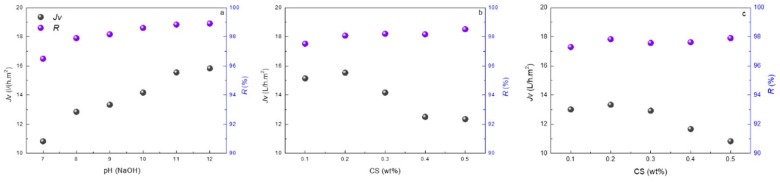
(**a**) Effects of NaOH concentration on membrane performance of PAUt membranes; Effects of CS concentration on membrane performance of (**b**) PAUt-CS and (**c**) PAUt-PDDA/PSS/CS. Testing conditions: 1.55 MPa and 2000 ppm NaCl as feed solution at 25.0 ± 2.0 °C.

**Figure 8:**
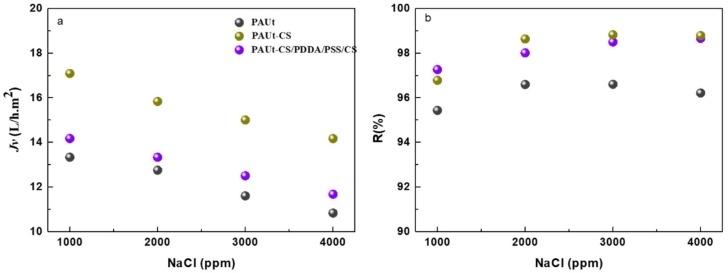
The reverse osmosis membrane performance under different salt concentration. (**a**) water flux and (**b**) salt rejection. Testing conditions: Applied pressure is 1.55 MPa with 1000–4000 ppm NaCl as feed solution at 25.0 ± 2.0 °C.

The modification conditions were optimized (Figure 7), and the reverse osmosis membrane performance was evaluated under different salt solution concentration (Figure 8). The modified PAUt-CS and PAUt-PDDA/PSS/CS membranes showed higher water flux and salt rejection than the nascent PAUt membrane. 

### 3.3. Fouling Property of the Membranes

**Figure 9:**
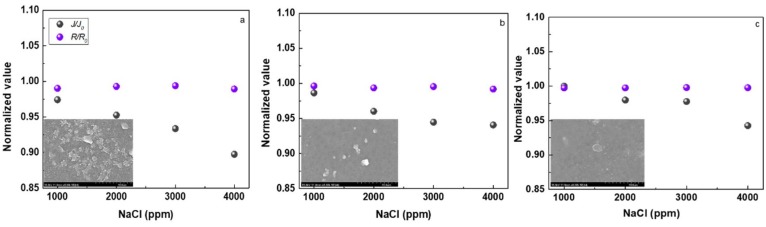
The normalized reverse osmosis performance of the fouled membrane with different salt concentration. (**a**) PAUt membranes; (**b**) PAUt-CS membranes and (**c**) PAUt-PDDA/PSS/CS membranes. Testing conditions: Applied pressure is 1.55 MPa with 1000–4000 ppm NaCl as feed solution at 25.0 ± 2.0 °C.

Fouling study was evaluated for the membrane after fouled by real lake water. The results showed that PAUt-PDDA/PSS/CS exhibited better antifouling performance than the modified PAUt-CS membrane and nascent PAUt membrane (Figure 9). 

Combining the reverse osmosis performance with the anti-fouling property as above showed, the PAUt-PDDA/PSS/CS membrane exhibited a promising candidate to be used in real applications. 

## 4. Discussion

### 4.1. Chemical Composition of the Membranes

The chemical composition of the resultant membrane selective separation layer was characterized by FTIR-ATR and XPS, respectively, while XPS is highly surface specific with a sampling depth of only 1–5 nm. The combination of FTIR-ATR and XPS results provided more information at different physical scales [38]. The FTIR-ATR spectra of PAUt, PAUt-CS and PAUt-PDDS/PSS/CS were presented in Figure 3. As FTIR-ATR scans could obtain the information from a relatively deeper surface (i.e., in micron level), information from both of the substrate and the selective separation layer was obtained [38]. In general, similar spectra over the 1000–4000 cm^−1^ range was exhibited for all the membrane likely due to the same polysulfone substrate and similar structure of PAUt with polyamide. For example, peaks at 1504 cm^−1^ and 1587 cm^−1^ were associated with the inherent spectrum from polysulfone [39,40]. In addition, the peak of 1609 cm^−1^ is the characteristic peaks for N‒H deformation vibration and C=C ring stretching vibration, 1663 cm^−1^ is associated with the C=C ring stretching vibration and N‒H deformation vibration in the –CO–NH– group and 1730 cm^−1^ is linked with the C=O in the –O–CO–NH– in the PAUt selective separation layer [34]. Interestingly, a wide peak appeared in 3389 cm^−1^, which may be associated with the hydrolyzation of –COCl to –COOH in CFIC monomer. However, no new fingerprint peaks could be observed after surface modification. A possible reason may be the overlapping of absorption bands: e.g., the –N–H– stretching band from CS is overlapped with that of –O–H– stretching. In addition, the –SO_3_ peak from PSS is overlapped with the peak from polysulfone substrate. 

The selective separation polyamide layer of PAUt membrane formed in interfacial polymerization is typically highly crosslinked, which is a key for high salt rejection. However, the reaction scheme and its variations also could happen at the same time. In Figure 4, four possible reaction schemes are proposed including one fully crosslinked reaction route and three possible linearly crosslinked reaction routes drawn with one repeat unit. In route A, the ideal structures of PAUt selective separation layer resulted from the full cross-linkage between MPD and CFIC; In route B, the hydrolysis of one –COCl group results in one pendant –COOH; In route C, the hydrolysis of the –OCOCl group results in one –OH group; In route D, the hydrolysis of one –OCOCl and one –COCl results in both –COOH and –OH groups with the molar ratio of 1:1. In each of the route, there is a specific element content according to the element ratio in each repeating unit. For example, the carbon content for route A (denoted as C% (A)), B (denoted as C% (B)), C (denoted as C% (C)) is 68.84%, 70.34%, and 64.02% respectively. The contents of N and O can be calculated using similar rationale. To facilitate discussion, the relative atomic concentration of C, O, and N, as well as the ratio of C/N, C/O, and O/N in the ideal structures, are summarized in Table 1. In reality, all three routes could happen at the same time, leading to a combination of the three units in the actual PAUt network. The portion of the units of A, B, C in a realistic PAUt network can be assumed as *x*, *y*, and *z*, where *x* + *y* + *z* = 1. Thus, the C, N, O content in a PAUt network can be represented as: C%(PAUt) = *x*C%(A) + *y*C%(B) + *z*C%(C), O%(PAUt) = *x*O%(A) + *y*O%(B) + *z*O%(C), and N%(PAUt) = *x*N%(A) + *y*N%(B) + *z*N%(C) respectively. Meanwhile, the quantitative composition of the elements from the PAUt selective separation layer (i.e., C%(PAUt), O%(PAUt), and N%(PAUt)), which has been determined by XPS elemental analysis, is also listed in Table 1. The value of *x*, *y*, and *z* can therefore be resolved to be 0.687, 0.195 and 0.1177 by substituting the value of real C% (PAUt), N% (PAUt), and O% (PAUt) into the equations above. This suggests the real PAUt network should contain 68.7% of unit A, 19.5% of unit B, and 11.8% of unit C. The quite abundance of units B and C ensure that there is a large quantity of available –COOH and –OH end groups for the stable electrical deposition of subsequent polyelectrolyte.

The O/N ratio of PAUt-CS (4.63) and PAUt-PDDA/PSS/CS (3.72) membrane surface is closer to the CS (4.57) (Scheme S1, Table S1) compared with the PAUt membrane (2.26), suggesting the successful assembly of CS thin layers on the surface of both membranes. At the same time, the O% (31.39 and 29.81, respectively) of both membranes is higher than the PAUt membrane (21.19). This likely resulted from the high O% in the CS molecules (42.64) (see Table S1) or the increased oxygen content during NaOH hydrolysis. Comparing the PAUt-CS and PAUt-PDDA/PSS/CS membrane, the decreased O% content and the increased N% content of the latter implies the successful assembly of PDDA as it has high N content and no O content in the molecular structure (Scheme S1, Table S1). From these results, we have learned that the PAUt membrane surface is successfully modified by CS molecules.

### 4.2. Morphology Characterization of the Membranes

SEM and AFM images of virgin and modified PAUt membranes are shown in Figure 5. In the first raw, PAUt membrane surface is characterized by the rough nodular structure, (Figure 5a), similar to the typical polymiade ridges and valley structures described in the literature [38]. After coating with CS (Figure 5b), a quite similar structure was resulted as compared with the virgin membrane. It indicates a rather thin CS layer coating has formed, which could be carried away by physical rinsing (the detail discussion will be presented in Section 3.3). Interestingly, distinct ridge and valley patterns almost disappeared for PAUt/PDDA/PSS/CS (Figure 5c). Thus, it can be inferred that the electrostatically assembled polyelectrolyte layers contributed to cover the rough surface and resulted in a relatively smooth surface. This observation can be confirmed by comparing the coated and virgin membranes in AFM images, where the root mean square (RMS) value of the PAUT/PDDA/PSS/CS is the lowest (i.e., 33.01 nm compared with the 74.22 nm for PAUt and 54.03 nm for PAUt/CS). The smoother surface will benefit better antifouling property, being a disc, which will be discussed in the later section.

### 4.3. Hydrophilicity of the Membranes

The water contact angle was used to evaluate the hydrophilicity of TFC membranes (Figure 6). In general, the membrane is hydrophilic at the contact angle θ less than 90°, while the membrane is hydrophobic at the contact angle θ more than 90°. The smaller the contact angle is, the higher the membrane hydrophilicity is. As shown in Figure 6, the water contact angles of PAUt-CS (46.2°) and PAUt-PDDA/PSS/CS (46.4°) are nearly the same, but they are obviously less than that of PAUt membrane (56.6°). In other words, coated-CS layer endows the two modified membranes with the same hydrophilicity, while results in much better hydrophilicity than the virgin membrane. This is probably because CS is a hydrophilic matter due to the existence of hydroxyl groups, which contributes greatly to the improvement of hydrophilicity of the membrane surface. It is well known that a high hydrophilic surface is an important characteristic in terms of good membrane permeability and anti-fouling property, which will be discussed in detail later.

### 4.4. Reverse Osmosis Performance of the Membranes

#### 4.4.1. The Effect of Fabrication Conditions on Membrane Separation Performance

The effect of NaOH treatment on the RO performance of PAUt membrane is shown in Figure 7a. 2000 ppm NaCl was used as feed solution under 1.55 MPa applied pressure. In general, higher water flux resulted from higher pH NaOH treatment because hydrolyzation of the PAUt selective separation layer creates a more hydrophilic surface. A similar trend was also observed for NaCl rejection. The possible reason may be the more negatively charged surface at high pH hydrolyzed surface contributes to ion rejection through Donnan exclusion mechanism. In addition, the more negatively charged surface may be favorable for CS assembly in the following step. Therefore, in the following study, the pH value of 12 was chosen to hydrolyze the PAUt selective separation layer.

The effects of CS concentration on the performance of PAUt-CS and PAUt-PDDA/PSS/CS membranes are shown in Figure 7b,c, respectively. Generally, the salt rejection tends to increase with the increase of CS concentration. Likely, the thicker CS coating layer on top of the polyamide layer eliminates some defects created in the IP process. At the same time, water flux steadily decreases at higher CS concentration range, likely caused by larger hydraulic resistance induced by thicker CS coating layer. Nonetheless, at lower CS concentration range, the water flux was found to increase with the increase of CS concentration up to 0.2%. This increase can be attributed to the better hydrophilicity of CS coated surface (i.e., the lower contact angle of PAUt-CS and PAUt-PDDA/PSS/CS than the PAUt membrane, also refer to Section 4.3) which favors the water transportation [20]. As the result of the trade-off between hydrophilicity and high resistance, the optimum concentration for CS was determined to be 0.2%.

#### 4.4.2. The Effect of Salt Solution Concentration on the Membrane Separation Performance

The water flux and salt rejection of PAUt, PAUt-CS and PAUt-PDDA/PSS/CS were shown in Figure 8. Various concentrations of NaCl solution were utilized in the current study. To get the stabilized water flux and salt rejection results, all the membranes were compacted under 1.55 MPa for at least two hours, and at least three independent membranes were tested and obtained the average results. Overall, the membrane water flux followed the sequence of PAUt < PAUt-CS < PAUt-PDDA/PSS/CS at all NaCl concentrations (Figure 8a). It should be noted, after NaOH hydrolyzation, the water flux exhibited around 16 liters per minute (LPM) at 2000 ppm NaCl solution. It was clearly observed that PAUt-CS had a similar water flux as compared with the NaOH hydrolyzed PAUt membranes, while showed a higher water flux as compared with the nascent PAUt membranes. After further coating with PDDA/PSS and caped with CS, the water flux significantly reduced due to more resistance from the two additional polyelectrolytes layers. However, the water flux of PAUt-PDDS/PSS/CS was still higher than the nascent PAUt membranes. With the increase of the feed solution concentration, water flux of all types of membranes reduced because of the decrease of effective hydraulic pressure. In addition to this, concentration polarization can add to the decrease of water flux at higher salt solution concentration [41]. The salt rejection against NaCl for different types of membranes was shown in Figure 8b. Generally, the salt rejection followed the order of PAUt-CS ≈ PAUt-PDDA/PSS/CS > PAUt. There are several possible reasons to lead the phenomenon: (1) the significantly lower water flux of PAUt leads to the lower rejection [42]; (2) the additional coating layer contributes to the increasing salt rejection of the two modified membranes as compare with the nascent PAUt membranes; (3) the additional coating layer of PDDA/PSS slightly changed the density of the active layer of PAUt-PDDA/PSS/CS membrane as compared with that of PAUt-CS, which lead to the similar salt rejection of PAUt-CS and PAUt-PDDA/PSS/CS.

### 4.5. Antifouling Property of the Membranes

The reverse osmosis membrane performance of all fouled membranes was evaluated under the same conditions as in Section 4.4. For all the membranes, the pre-filtered real lake water was used as feed solution and the operation was continued for seven days. The normalized water flux and salt rejection with different salt solution concentration were presented in Figure 9. The normalized value of 1 indicates no change of water flux or rejection happened after membrane soaking in the real lake water, where J/J_0_ indicates the ratio of water flux after fouling and before fouling, respectively. Similarly, R/R_0_ indicates the ratio of rejection after fouling and before fouling, respectively.

Clearly, PAUt-PDDA/PSS/CS exhibited a higher normalized value of water flux than the PAUt-CS as well as the PAUt membranes. It is easily understood that a smooth surface and a hydrophilic surface contribute to the lower fouling property. In addition, the SEM of fouled images showed that much fewer foulants deposited on the membrane surface for the PAUt-PDDA/PSS/CS. However, the foulants have more impact on the water flux than the rejection. The normalized value of rejection is closed to 1 for all types of membranes. Interestingly, with the increase of the salt solution concentration, more severe fouling issue happened. This may be indicated the more complex mechanisms happened among salt and the foulants, therefore resulted in the lower water flux. 

## 5. Conclusions

The polyamide-urethane (PAUt) membranes were successfully fabricated via MPD and CFIC monomers, and then their PAUt selective separation layers were further modified for antifouling purpose without scarifying the reverse osmosis performance via two modification methods. The modified PAUt-CS and PAUt-PDDA/PSS/CS membranes not only had smoother and more hydrophilic surface but also showed higher water flux and salt rejection than the nascent PAUt membrane. Especially, the PAUt-PDDA/PSS/CS membrane exhibited the better antifouling performance than the others. Combining the reverse osmosis performance with the anti-fouling property, obviously, the PAUt-PDDA/PSS/CS membrane behaved as a promising candidate to be used in real applications.

## Figures and Tables

**Table 1:** Elemental composition of the possible ideal structure and the actual data of XPS analysis for PAUt membranes.

Membrane	Structure	C%	O%	N%	C/N	C/O	O/N
PAUt	Fully crosslinked (A)	68.84	18.80	12.36	5.55	3.66	1.52
	Linear crosslinked (B)	70.34	18.73	10.93	6.44	3.76	1.71
	Linear crosslinked (C)	64.02	26.65	9.33	6.86	2.40	2.86
PAUt	Actual	69.44	21.19	9.37	7.41	3.28	2.26
PAUt-CS	Actual	61.83	31.39	6.78	9.12	1.97	4.63
PAUt-PDDA/PSS/CS	Actual	62.19	29.81	8.01	7.76	2.08	3.72

## Supplementary Materials

The following are available online at http://www.mdpi.com/2073-4360/10/4/346/s1, Scheme S1: Chemical structure of the used polyelectrolytes, Table S1: Elemental composition of the used polyelectrolytes. Table S2: Composition of the West Lake water after microfiltration.

## References

[1] Elimelech M., Phillip W.A. (2011). The future of seawater desalination: Energy, technology, and the environment. Science.

[2] Malaeb L., Ayoub G.M. (2011). Reverse osmosis technology for water treatment: State of the art review. Desalination.

[3] Song X., Qi C., Tang C., Gao C. (2017). Ultra-thin, multi-layered polyamide membranes: Synthesis and characterization. J. Membr. Sci..

[4] Kang G., Cao Y.M. (2012). Development of antifouling reverse osmosis membranes for water treatment: A review. Water Res..

[5] Fujiwara N., Matsuyama H. (2008). Elimination of biological fouling in seawater reverse osmosis desalination plants. Desalination.

[6] Bartman A.R., Lyster E., Rallo R., Christofides P.D. (2011). Mineral scale monitoring for reverse osmosis desalination via real-time membrane surface image analysis. Desalination.

[7] Weinman T.S., Husson S.M. (2016). Influence of chemical coating combined with nanopatterning on alginate fouling during nanofiltration. J. Membr. Sci..

[8] Hong A.N.T., Tran D.T., Dinh C.H. (2017). Surface photochemical graft polymerization of acrylic acid onto polyamide thin film composite membranes. J. Appl. Polym. Sci..

[9] Zhou Y., Yu S., Gao C., Feng X. (2009). Surface modification of thin film composite polyamide membranes by electrostatic self deposition of polycations for improved fouling resistance. Sep. Purif. Technol..

[10] Wang N., Liu T., Shen H., Ji S., Li J.R., Zhang R. (2016). Ceramic tubular MOF hybrid membrane fabricated through in situ layer-by-layer self-assembly for nanofiltration. AIChE J..

[11] Wang L., Wang N., Li J., Li J., Bian W., Ji S. (2016). Layer-by-layer self-assembly of polycation/GO nanofiltration membrane with enhanced stability and fouling resistance. Sep. Purif. Technol..

[12] Chen Q., Yu P., Huang W., Yu S., Liu M., Gao C. (2015). High-flux composite hollow fiber nanofiltration membranes fabricated through layer-by-layer deposition of oppositely charged crosslinked polyelectrolytes for dye removal. J. Membr. Sci..

[13] Zhao J., Pan F., Li P., Zhao C., Jiang Z. (2013). Fabrication of Ultrathin Membrane via Layer-by-Layer Self-assembly Driven by Hydrophobic Interaction towards High Separation Performance. ACS Appl. Mater. Interfaces.

[14] Zhang G., Yan H., Ji S., Liu Z. (2007). Self-assembly of polyelectrolyte multilayer pervaporation membranes by a dynamic layer-by-layer technique on a hydrolyzed polyacrylonitrile ultrafiltration membrane. J. Membr. Sci..

[15] Kotov N.A., Magonov S., Tropsha E. (1998). Layer-by-layer self-assembly of alumosilicate-polyelectrolyte composites: Mechanism of deposition, crack resistance, and perspectives for novel membrane materials. Chem. Mater..

[16] Lin H., Zhao C., Ma W., Li H., Na H. (2009). Layer-by-layer self-assembly of in situ polymerized polypyrrole on sulfonated poly(arylene ether ketone) membrane with extremely low methanol crossover. Int. J. Hydrog. Energy.

[17] Sun G.F., Sun G., Chung T.S., Jeyaseelan K., Armugam A. (2013). A layer-by-layer self-assembly approach to developing an aquaporin-embedded mixed matrix membrane. RSC Adv..

[18] Cho K.L., Cho K.L., Hill A.J., Caruso F., Kentish S.E. (2015). Chlorine Resistant Glutaraldehyde Crosslinked Polyelectrolyte Multilayer Membranes for Desalination. Adv. Mater..

[19] Qin Z.P., Ren X., Shan L., Guo H., Geng C., Zhang G., Ji S., Liang Y. (2016). Nacre like-structured multilayered polyelectrolyte/calcium carbonate nanocomposite membrane via Ca-incorporated layer-by-layer-assembly and CO_2_-induced biomineralization. J. Membr. Sci..

[20] Saren Q., Qiu C.Q., Tang C.Y. (2011). Synthesis and characterization of novel forward osmosis membranes based on layer-by-layer assembly. Environ. Sci. Technol..

[21] Qi S., Li Y., Wang R., Tang C.Y. (2016). Towards improved separation performance using porous FO membranes: The critical roles of membrane separation properties and draw solution. J. Membr. Sci..

[22] Qi S., Li W., Zhao Y., Ma N., Wei J., Chin T.W. (2012). Influence of the properties of layer-by-layer active layers on forward osmosis performance. J. Membr. Sci..

[23] Xu J., Feng X., Gao C. (2011). Surface modification of thin-film-composite polyamide membranes for improved reverse osmosis performance. J. Membr. Sci..

[24] Liu L.F., Yu S.C., Wu L.G., Gao C.J. (2008). Study on a novel antifouling polyamide-urea reverse osmosis composite membrane (ICIC-MPD): III. Analysis of membrane electrical properties. J. Membr. Sci..

[25] Li L., Zhang S., Zhang X., Zheng G. (2007). Polyamide thin film composite membranes prepared from 3,4',5-biphenyl triacyl chloride, 3,3′,5,5′-biphenyl tetraacyl chloride and m-phenylenediamine. J. Membr. Sci..

[26] Louie J.S., Pinnau I., Ciobanu I., Ishida K.P., Ng A., Reinharda M. (2006). Effects of polyether-polyamide block copolymer coating on performance and fouling of reverse osmosis membranes. J. Membr. Sci..

[27] Liu L.F., Mao P.Q., Zhi X., Zhang L., Gao C.J. (2012). Chemical Structure and Performance of a Novel Polyimide-urethane Composite Reverse Osmosis Membrane Material. Chem. J. Chin. Univ. Chin..

[28] Rana D., Kim Y., Matsuura T., Arafat H.A. (2011). Development of antifouling thin-film-composite membranes for seawater desalination. J. Membr. Sci..

[29] Yin J., Zhu G.C., Deng B.L. (2016). Graphene oxide (GO) enhanced polyamide (PA) thin-film nanocomposite (TFN) membrane for water purification. Desalination.

[30] Jeong B.-H., Hoek E.M.V., Yan Y., Subramani A. (2007). Interfacial polymerization of thin film nanocomposites: A new concept for reverse osmosis membranes. J. Membr. Sci..

[31] Kim S.J., Lee P.S., Bano S., Park Y.I. (2016). Effective incorporation of TiO2 nanoparticles into polyamide thin-film composite membranes. J. Appl. Polym. Sci..

[32] Tang C.Y., Wang Z., Petrinić I., Fane A.G., Hélix-Nielsen C. (2015). Biomimetic aquaporin membranes coming of age. Desalination.

[33] Park S.-H., Ko Y.S., Park S.J., Lee J.S., Cho J. (2016). Immobilization of silver nanoparticle-decorated silica particles on polyamide thin film composite membranes for antibacterial properties. J. Membr. Sci..

[34] Liu L.F., Cai Z.B., Shen J.N., Wu L.X., Hoek E.M.V. (2014). Fabrication and characterization of a novel poly (amide-urethane@imide) TFC reverse osmosis membrane with chlorine-tolerant property. J. Membr. Sci..

[35] Qi S., Li Y., Zhao Y., Li W., Tang C.Y. (2015). Highly efficient forward osmosis based on porous membranes-Applications and implications. Environ. Sci. Technol..

[36] Qiu C., Qi S., Tang C.Y. (2011). Synthesis of high flux forward osmosis membranes by chemically crosslinked layer-by-layer polyelectrolytes. J. Membr. Sci..

[37] Qi S., Wang R., Chaitra G.K.M., Torres J., Hu X. (2016). Aquaporin-based biomimetic reverse osmosis membranes: Stability and long term performance. J. Membr. Sci..

[38] Tang C.Y., Kwon Y.N., Leckie J.O. (2007). Probing the nano-and micro-scales of reverse osmosis membranes-A comprehensive characterization of physiochemical properties of uncoated and coated membranes by XPS, TEM, ATR-FTIR, and streaming potential measurements. J. Membr. Sci..

[39] Kwon N.Y., Leckie J.O. (2006). Hypochlorite degradation of crosslinked polyamide membranes. II. Changes in hydrogen bonding behavior and performance. J. Membr. Sci..

[40] Tang C.Y., Kwon Y., Leckie J.O. (2009). Effect of membrane chemistry and coating layer on physiochemical properties of thin film composite polyamide RO and NF membranes: I. FTIR and XPS characterization of polyamide and coating layer chemistry. Desalination.

[41] Fane A.G., Tang C.Y., Wang R. (2011). Membrane Technology for Water: Microfiltration, Ultrafiltration, Nanofiltration, and Reverse Osmosis.

[42] Geise G.M., Park H.B., Sagle A.C., Freeman B.D., Mcgrath J.E. (2011). Water permeabilet ality and water/salt selectivity trade off in polymers for desalination. J. Membr. Sci..

