# Supplementary material for: Modification of Polyamide-Urethane (PAUt) Thin Film Composite Membrane for Improving the Reverse Osmosis Performance
Source: Polymers (Basel). 2018 Mar 21;10(4):346. doi: 10.3390/polym10040346 (PMC6415036; doi:10.3390/polym10040346)
Supplement: Supplementary file 1 [file polymers-10-00346-s001.pdf]

Supporting Information

Modification of polyamide-urethane (PAUt) thin film composite membrane for improving the reverse osmosis performance

Li-Fen Liu[1,2*], Xing-Ling Gu[1], Sa-Ren Qi[1,2],Xin Xie[1], Rui-Han Li[1], Ke Li[3], Chun-Yang Yu[3*], Cong-Jie Gao[1,2]

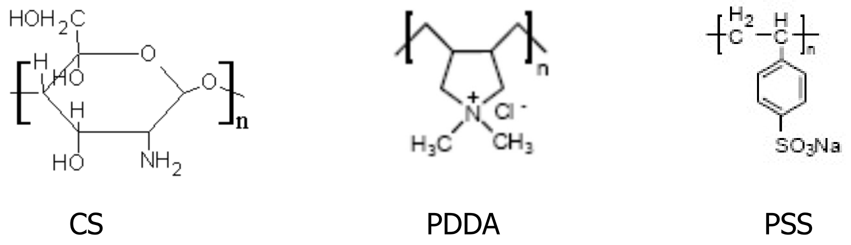

Scheme 1 Chemical structure of the used polyelectrolytes

Table 1 Elemental composition of the used polyelectrolytes

Polyelectrolyte	C%	O%	N%	C/N	C/O	O/N
CS	48.02	42.64	9.34	5.14	1.12	4.57
PDDA	87.28	0	12.72	6.86	/	0
PSS	54.54	27.25	0	/	2.00	/

Table S2 Composition of the West Lake water after microfiltration

Species	Quantity
K^+ and Na^+ , mg/l	12.0
Ca^{2+} , mg/l	30.8
Mg^{2+} , mg/l	2.3
$Fe_{(Total)}$,mg/l	0.03

HCO_3^- , mg/l	79.6
Cl^- , mg/l	20.0
SO_4^{2-} , mg/l	18.4
HA , mg/l	<5
NO_2^- , mg/l	10.8
SiO_2 , mg/l	8.0
Turbidity, NTU	0.8
$COD_{(Mn)}$, mg/l	4.3
Bacteria, CFU/ml	<150
